# Emotional blunting in patients with depression. Part I: clinical characteristics

**DOI:** 10.1186/s12991-022-00387-1

**Published:** 2022-04-04

**Authors:** Michael Cronquist Christensen, Hongye Ren, Andrea Fagiolini

**Affiliations:** 1 Medical Affairs, H. Lundbeck A/S, Ottiliavej 9, 2500 Valby, Denmark; 2 Division of Psychiatry, Department of Molecular and Developmental Medicine, University of Siena School of Medicine, Siena, Italy

**Keywords:** Depression, Emotional blunting, Functioning, Oxford Depression Questionnaire, Patient perspectives, Recovery

## Abstract

**Background:**

Emotional blunting—inability to feel positive or negative emotions, detachment, or reduced emotional responsiveness—is common in people with depression. However, there is a paucity of studies comprehensively investigating this symptom and its functional impact. This study investigated the experience of emotional blunting, and its impact on overall functioning and quality of life, in the acute and remission phases of depression from the perspective of patients and healthcare providers. This paper presents data on the clinical presentation of emotional blunting in depression from the patient perspective.

**Methods:**

Cross-sectional, observational study conducted in Brazil, Canada, and Spain between April 15 and May 18, 2021. Data were collected via a self-completed online survey. Respondents were adults with depression (acute or remission phase), who were currently using a prescribed antidepressant, and who reported emotional blunting during the past 6 weeks. Emotional blunting was assessed using the Oxford Depression Questionnaire (ODQ; total score range 26–130, higher scores indicate greater emotional blunting).

**Results:**

In all, 752 patients completed the survey (62% female; mean age, 45 years). Overall, 44% of patients rated their emotional blunting as extremely severe (acute phase [*n* = 300], 72%; remission phase [*n* = 452], 25%; difference, *p* < 0.01). In all, 56% of patients considered their emotional blunting to be caused by their depression (acute phase, 62%; remission phase, 52%). Mean ODQ total score was 94.8 for patients in the acute phase of depression and 85.7 for those in remission (difference, *p* < 0.01). Mean score for the ODQ ‘antidepressant as cause’ domain (maximum possible score, 30) was 18.0 in patients in the acute phase and 17.6 in those in remission. Overall, 45% of patients believed that their antidepressant medication was blunting their emotions and 39% were considering stopping or had already stopped their antidepressant because of perceived emotion-related side effects.

**Conclusions:**

Almost three-quarters of patients in the acute phase of depression and one-quarter of those in remission reported severe emotional blunting. Approximately 56% of patients considered their emotional blunting to be caused by their depression, while 45% believed that their antidepressant medication was negatively affecting their emotions. Just over one-third of patients were considering stopping or had stopped their antidepressant as a result.

**Supplementary Information:**

The online version contains supplementary material available at 10.1186/s12991-022-00387-1.

## Background

Depression is a common mental health disorder, affecting more than 264 million individuals worldwide and contributing greatly to the overall global burden of disease [1]. Compared with the general population, patients with depression have impaired functioning across multiple domains, and this impacts significantly on their work, family, and social lives [2, 3]. Functional recovery is therefore an important treatment goal in patients with depression [4–7].

Emotional blunting—usually defined as the inability to feel positive or negative emotions; feelings of detachment; or reduced emotional responsiveness—is a common symptom in patients with depression, including those receiving selective serotonin reuptake inhibitors (SSRIs) or serotonin–noradrenaline reuptake inhibitors (SNRIs) [8–14]. In a survey of more than 1800 patients in New Zealand taking a range of different antidepressants, 60% of respondents reported feeling emotionally numb [10]. Emotional blunting is also a common reason for discontinuing antidepressant therapy in patients with depression [15].

There is, however, a paucity of studies comprehensively investigating this symptom and its potential impact on patients’ overall functioning and health-related quality of life. The present study was undertaken in order to address this data gap. The study aimed to explore the lived experience of emotional blunting in patients with major depressive disorder (MDD), and its impact on overall functioning and health-related quality of life, in both the acute and remission phases of MDD from the perspective of patients and healthcare providers. This paper—the first in a series exploring different aspects of emotional blunting in MDD—presents data on the clinical characteristics of emotional blunting from the patient perspective. Subsequent papers in the series will explore the impact of emotional blunting on functioning and overall quality of life, the prevalence of trauma and its association with emotional blunting, and differences between patient and healthcare provider perspectives regarding the experience of emotional blunting in patients with MDD.

## Methods

### Participants

Eligible patients were aged 18–70 years, had been diagnosed with depression by a physician, were currently using a prescribed antidepressant, and reported having experienced emotional blunting during the last 6 weeks. Enrollment quotas were imposed with respect to patient age (50% aged ≥ 50 years) and sex (60% female). Emotional blunting was defined using a validated screening question [16]: ‘*Emotional effects of depression and treatment vary, but may include, for example, feeling emotionally “numbed” or “blunted” in some way; lacking positive emotions or negative emotions; feeling detached from the world around you; or ‘just not caring’ about things that you used to care about. Have you experienced such emotional effects during the last 6 weeks?’* Patients were also required to be in either the acute or remission phase of depression. Acute phase was defined as: *‘A time when your symptoms are at their worst or most severe and for which you use antidepressant treatment.’* Remission phase was defined as: *‘A time when your symptoms have improved significantly and you are already feeling better, but you may or may not still experience some minor symptoms. You are still taking antidepressant medication.’*

Patients who responded ‘No’ to the screening question for emotional blunting or who were not in the acute or remission phase of depression were excluded from study participation. Patients currently employed by a pharmaceutical company or market research agency were also excluded.

### Study design

This was a cross-sectional, observational study conducted by BPR Pharma (London, UK) in Brazil, Canada, and Spain between April 15 and May 18, 2021. Data were collected by means of a self-completed online survey. The approximate time required for patients to complete the survey was 25 min. The survey primarily comprised closed questions encompassing semantic rating scales, but also included some open questions to capture verbatim responses. The survey questionnaires were pre-tested and refined by undertaking five pilot interviews in Canada. Following this pre-test, some minor refinements to the questionnaires were implemented. The final questionnaires were translated into local languages.

Recruitment of study participants, scripting of the online survey questionnaires, and hosting were undertaken by Kantar (London, UK). A random selection of potential participants were identified from Kantar’s online panel of consumers and invited to participate. Potential respondents were provided with information about the study before being screened for eligibility. Participants had previously consented to participate in research. Informed consent was also obtained from all respondents specifically for this study prior to screening. At the beginning of the survey, participants were informed that they could refuse to answer any question and could withdraw from participation at any point. Respondents were rewarded for their participation in the study.

The study and all associated materials were approved by an institutional review board prior to initiation (Veritas IRB, Montreal, QC, Canada). The study was conducted in accordance with the European Pharmaceutical Market Research Association (EphMRA) code of conduct, which ensures respondent confidentiality, and adhered to General Data Protection Regulation (GDPR) and all local market laws regarding data protection.

### Assessments

Respondents were asked which of a list of symptoms they had experienced during their current phase of depression (including emotional blunting), and were asked to rate the severity of the symptoms experienced on a scale of 1 (not at all severe) to 7 (extremely severe). The online survey also incorporated the Oxford Depression Questionnaire (ODQ) [16, 17], the Functioning Assessment Short Test [18], the World Health Organization-Five Well-being Index [19], and trauma questions based on the Childhood Trauma Questionnaire [20].

The ODQ is a validated instrument for assessing emotional blunting in patients with depression, including those treated with antidepressants [16, 17]. It comprises 26 questions about emotional experiences during the past week, for which respondents are asked the extent to which they agree or disagree. Questions cover five domains of emotional blunting: general reduction in emotions, reduction in positive emotions, emotional detachment from others, not caring, and antidepressant as cause. The ‘antidepressant as cause’ domain is only completed by patients currently receiving antidepressants for their depression, and explores the respondent’s perception of a potential link between their current antidepressant and their experience of emotional blunting, and whether this has affected their adherence to treatment. For each question, responses are indicated on a 5-point scale ranging from 1 (disagree) to 5 (agree). An overall ODQ score is computed, as well as scores for each ODQ domain. All domains are scored out of 25, except for the ‘antidepressant as cause’ domain (scored out of 30). The ODQ total score ranges from 26 to 130 points, with higher scores indicating more severe emotional blunting.

### Statistical analysis

The population for this analysis comprised all patients who met the study inclusion criteria and completed the online survey. Data were analyzed by The Stats People (Sevenoaks, UK) using MERLIN tabulation software and Microsoft Excel. Data are presented descriptively using means and standard deviations (SDs) for continuous variables, and frequencies and percentages for categorical variables. Comparisons were performed using *t* tests for continuous measures and Z tests for proportions; significance was set at *p* < 0.05.

## Results

### Patient demographics

Of 5127 patients invited to participate, 3985 were excluded as they did not meet the inclusion criteria and 245 were excluded due to quota requirements; a further 145 patients were excluded as they did not complete the survey. A total of 752 patients completed the survey (approximately equal numbers from Brazil, Canada, and Spain). Patient demographics and baseline characteristics are shown in Table 1. In all, 40% of patient respondents indicated that they were in the acute phase of depression and 60% that they were in remission. Patient demographics and baseline characteristics were generally similar between patients in the acute and remission phases. However, a significantly greater proportion of patients in the remission phase of depression were in full-time employment (50% vs 40% in the acute phase; *p* < 0.01) and a significantly greater proportion in the acute phase of depression reported a history of any severe trauma (92% vs 85% in the remission phase; *p* < 0.01). Most patients were receiving treatment with SSRIs or SNRIs, most commonly fluoxetine (26% overall; 29% in the acute phase group and 25% in the remission phase group).

**Table 1:** Patient demographics and baseline characteristics, overall and by phase of depression

	All patients (*N* = 752)	Acute (*n* = 300)	Remission (*n* = 452)
Sex, *n* (%)			
Female	466 (62)^a^	195 (65)^a^	271 (60)^a^
Age, years			
Mean (SD)	45 (12)	46 (12)	47 (13)
Time since diagnosis of depression (months)			
Mean (SD)	62.4 (49.5)	61.3 (48.3)	63.2 (50.3)
High school education or above, n (%)	596 (79)	235 (78)	361 (80)
Work status, n (%)			
Full-time	348 (46)	120 (40)	228 (50)**
Part-time	93 (12)	33 (11)	60 (13)
In a relationship, n (%)	471 (63)	181 (60)	290 (64)
Ever addicted to drugs or alcohol, n (%)	156 (21)	71 (24)	85 (19)
Any severe trauma, n (%)	658 (88)	276 (92)**	382 (85)
Current treatment used for depression, n (%)^b^			
Fluoxetine	199 (26)	87 (29)	112 (25)
Escitalopram	125 (17)	52 (17)	73 (16)
Sertraline	121 (16)	53 (18)	68 (15)
Citalopram	110 (15)	48 (16)	62 (14)
Venlafaxine	100 (13)	46 (15)	54 (12)
Bupropion	79 (11)	46 (15)**	33 (7)
Paroxetine	78 (10)	32 (11)	46 (10)
Duloxetine	56 (7)	26 (9)	30 (7)
Mirtazapine	44 (6)	18 (6)	26 (6)
Desvenlafaxine	34 (5)	16 (5)	18 (4)
Vortioxetine	27 (4)	13 (4)	14 (3)
Agomelatine	26 (3)	15 (5)	11 (2)
Other drug therapy	176 (23)	84 (28)*	92 (20)
Unsure/don’t know	14 (2)	5 (2)	9 (2)

^a^A quota of 60% female was set in the study design

^b^Patients may have received more than one drug therapy

Difference between acute and remission phase groups, *p < 0.05; **p < 0.01

### Symptoms and severity

**Fig. 1:**
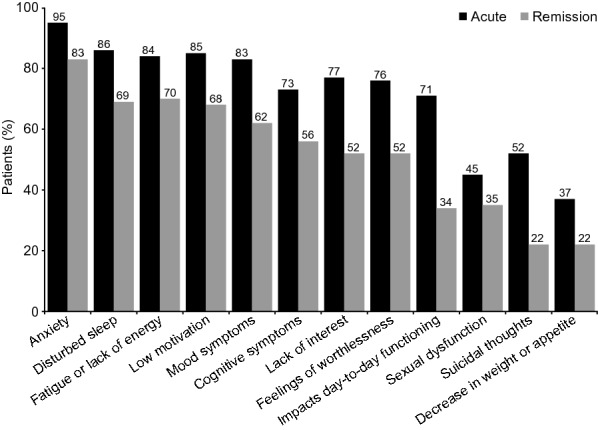
Proportion of patients in acute phase of depression (*n* = 300) or in remission (*n* = 452) reporting symptoms (*p* < 0.01 for all differences between the acute and remission phases)

Symptoms reported by patients according to their phase of depression are shown in Fig. 1. Irrespective of the phase of depression, anxiety was the most frequently experienced symptom (reported by 95% of patients in the acute phase of depression and 83% of those in the remission phase). In the acute phase of depression, sleep problems, fatigue/lack of energy, low motivation, and mood symptoms were all reported by more than 80% of patients. Lack of interest (i.e., anhedonia) was reported by 77% of patients in the acute phase of depression and 52% of those in the remission phase.

**Fig. 2:**
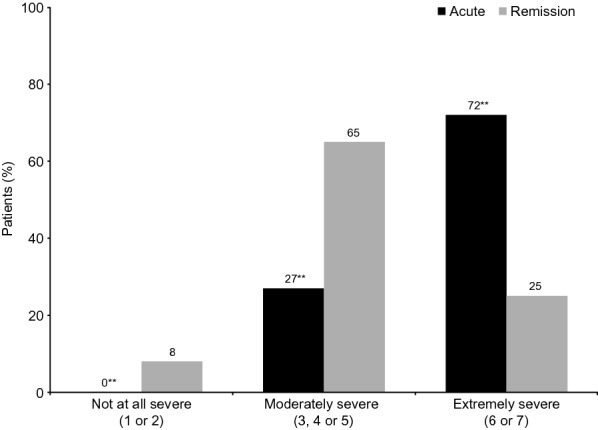
Severity of emotional blunting by phase of depression. **p < 0.01 for difference between the acute and remission phase groups

Per protocol, all patients had experienced emotional blunting within the past 6 weeks; their mean (SD) emotional blunting severity score was 5.2 (1.4) (maximum possible score, 7). Overall, 44% of patients rated their emotional blunting as extremely severe (i.e., a score of 6 or 7). Emotional blunting was more severe in patients in the acute phase of depression than in those in remission (Fig. 2). Extremely severe emotional blunting was reported by 72% of patients in the acute phase compared with 25% of those who were in remission (*p* < 0.01). Most patients considered emotional blunting to be caused by their depression (62% of patients in the acute phase of depression and 52% of those in remission; *p* < 0.01). A further 27% of patients in both disease phases believed that both their depression and their antidepressant medication contributed to their emotional blunting. Only 6% of patients in the acute phase of depression and 10% of those in remission considered their emotional blunting to be due to their antidepressant medication alone.

### ODQ scores

The mean (SD) patient-reported ODQ total score was 89.3 (18.3) (maximum possible score, 130) in the overall study population, 94.8 (16.6) in patients in the acute phase of depression, and 85.7 (18.4) in those in the remission phase (difference between phase subgroups, *p* < 0.01). The mean (SD) ODQ total score was significantly higher in men than women (93.5 [16.1] vs 86.8 [19.1] points, respectively; *p* < 0.01). There was no difference in mean (SD) ODQ total score between countries (89.9 [18.3] in Brazil, 88.4 [18.4] in Canada, and 89.6 [18.2] in Spain). Mean (SD) ODQ total score was significantly higher in patients who reported a history of addiction (93.3 [17.7] vs 88.3 [18.3] in those with no history of addiction; *p* < 0.01) and in patients who reported having experienced any severe trauma (90.1 [18.3] vs 83.9 [17.3] in those who did not; *p* < 0.01).

Irrespective of the phase of depression, the greatest contribution to the ODQ total score was from the ‘reduction in positive emotions’ domain and the least from the ‘emotional detachment’ domain (Table 2). The overall proportion of patients agreeing with each of the constituent statements in the ‘reduction in positive emotions’ domain ranged from 74 to 82% (Additional file 1: Table S1). A similar high level of patient agreement was seen for the following statements in the ‘general reduction in emotions’ domain: *‘Day-to-day life just doesn’t have the same emotional impact on me that it did before my illness/problem’* (75%) and ‘*My emotions are numbed/dulled/flattened compared to before I developed my illness/problem’* (72%). In the ‘not caring’ domain, 72% of patients agreed with the statement: *‘I feel “spaced out” and distant from the world around me.’*

**Table 2:** Mean (SD) ODQ domain scores and ODQ total score in the overall patient sample and according to phase of depression

ODQ domain^a^	Overall (*N* = 752)	Acute (*n* = 300)	Remission (*n* = 452)
Reduction in positive emotions	20.4 (4.3)	22.3 (3.3)**	19.2 (4.5)
General reduction in emotions	18.1 (3.8)	18.7 (4.0)**	17.7 (3.7)
Not caring	17.6 (4.7)	19.4 (4.3)**	16.3 (4.5)
Emotional detachment from others	15.4 (5.8)	16.3 (6.1)**	14.8 (5.5)
Antidepressant as cause	17.8 (6.1)	18.0 (6.4)	17.6 (5.9)
Total score	89.3 (18.3)	94.8 (16.6)**	85.7 (18.4)

^a^For all domains except ‘antidepressant as cause’, the maximum score is 25; for the ‘antidepressant as cause’ domain, the maximum score is 30. ODQ total score ranges from 26 to 130 points, and higher scores indicate more severe emotional blunting

^**^p < 0.01 for difference between the acute and remission phase groups

ODQ, Oxford Depression Questionnaire; SD, standard deviation

The proportion of patients agreeing with each of the constituent statements was significantly higher in the acute phase of depression than in the remission phase for both the ‘reduction in positive emotions’ domain (85–93% vs 66–74%, respectively; all *p* < 0.01) and the ‘not caring’ domain (59–83% vs 35–65%, respectively; all *p* < 0.01) (Additional file 1: Table S1). The proportion of patients agreeing with each statement in the ‘emotional detachment’ domain was also higher in the acute phase group than in the remission group, but was statistically significant only for ‘*I care less about other people’s feelings than I think I should’* (54% vs 45%; *p* < 0.05). In the ‘general reduction in emotions’ domain, significant differences between the two depression phase groups were seen for the following statements: ‘*Day-to-day life just doesn’t have the same emotional impact on me …’* (83% in the acute phase group vs 70% in the remission group; *p* < 0.01); ‘*My emotions are numbed/dulled/flattened …’* (77% vs 68%, respectively; *p* < 0.01); and *‘My emotions lack intensity’* (63% vs 54%, respectively; *p* < 0.05).

The mean (SD) ODQ score for the ‘antidepressant as cause’ domain was 18.0 (6.4) points in patients in the acute phase of depression and 17.6 (5.9) points in those in remission (maximum possible score, 30 points). The ODQ total score and all individual ODQ domain scores (with the exception of the ‘antidepressant as cause’ domain) were significantly higher in the acute phase group than in the remission phase group (all *p* < 0.01) (Table 2). Based on responses to the individual questions in the ‘antidepressant as cause’ domain (Table 3), 45% of patients indicated that they believed that their antidepressant medication was affecting their emotions, and 39% indicated that they were considering stopping or had already stopped their antidepressant because of perceived emotional side effects. The proportions of patients agreeing with each statement in the ‘antidepressant as cause’ domain were similar in the acute and remission phase groups (Table 3).

**Table 3:** Antidepressant as cause of emotional blunting as assessed by the ODQ

	Patient agreement^a^ (%)		
	All patients (*N* = 752)	Acute (*n* = 300)	Remission (*n* = 452)
The AD is preventing me from feeling my emotions in some way	45	46	44
The AD seems to make me just not care about things that should matter to me	40	40	40
The AD seems to make me feel emotionally disconnected from people around me	42	45	40
The AD is preventing me from feeling pleasant emotions	34	35	33
The AD changes the way that I experience my emotions in a way that is unhelpful (not helpful) to me at the moment	33	35	32
I have considered stopping (or have already stopped) my antidepressant because of its emotional side effects	39	39	40

^a^Proportion of patients who selected ‘agree’ or ‘agree a little’ in response to the statement

AD, antidepressant; ODQ, Oxford Depression Questionnaire

## Discussion

In this population of patients with MDD reporting emotional blunting, almost three-quarters of patients in the acute phase of depression rated their emotional blunting as extremely severe, and one-quarter of patients who were in remission from their depression continued to report extremely severe emotional blunting. Just over half of patients considered their emotional blunting to be caused by their depression, while 45% believed that their antidepressant medication was blunting their emotions. Of note, just over one-third of all patients were considering or had already stopped taking their antidepressant because of perceived emotion-related adverse effects.

Severity of emotional blunting was also evaluated using the ODQ. A recent study has demonstrated that this validated assessment scale captures aspects of emotional blunting in patients with depression receiving antidepressant treatment that are not well measured by other scales more commonly used for assessing depressive symptom severity [17]. The overall mean ODQ total score was 89.3 out of a possible maximum 130 points, reflecting the relatively high proportion of patients who rated their emotional blunting as extremely severe. This value is remarkably similar to that reported in patients with depression who had an inadequate response to an SSRI or SNRI and were experiencing emotional blunting on treatment in another recent study (mean ODQ score at baseline, 89.4 points) [21]. In the present study, patients with higher ODQ scores (i.e., more severe emotional blunting) were more likely to be in the acute phase of depression, to be male, to currently have addiction or have a history of addiction, and to have experienced severe trauma. Irrespective of the phase of depression, reduction in positive emotions was found to be the biggest contributor to the overall ODQ score.

It has been suggested that emotional blunting may be an adverse effect associated with certain classes of antidepressants; for example, up to 60% of patients treated with SSRIs or SNRIs report some degree of emotional blunting [8–11]. SSRIs and SNRIs were the most commonly prescribed classes of antidepressants for patients participating in the present study. Indeed, just under half of all patients believed that their antidepressant medication was blunting their emotions and more than one-third were considering stopping or had already stopped their antidepressant because of perceived emotion-related side effects. These findings are in line with the results of a previous online survey undertaken to identify determinants of treatment effectiveness and tolerability in patients with MDD or bipolar disorder; in that study, emotional blunting was found to be a common treatment-emergent adverse event, leading to discontinuation of antidepressant medication in more than one-third of respondents [15].

Our findings also highlight the broad range of symptoms experienced by patients with depression during both the acute and remission phases of the disease. The clinical presentation of depression is known to be highly heterogeneous, with considerable variation in the specific symptoms experienced among patients [22–25]. In another recent study undertaken to explore patients’ lived experiences of depression and their expectations for antidepressant therapy, the most frequent symptoms reported were fatigue (mentioned by 59% of respondents), lack of motivation/loss of interest (52%), anxiety/panic (45%), sadness (41%), and lack of concentration/brain fog (41%) [26].

Anhedonia (i.e., the inability to experience pleasure) is a common symptom of depression and other mental health disorders [8, 16]. The Diagnostic and Statistical Manual of Mental Disorders, fifth edition (DSM-5) recognizes anhedonia as one of the core diagnostic criteria for a major depressive episode [27]. Patients with emotional blunting very frequently experience anhedonia; however, the emotions that are blunted are not limited to pleasure, and other emotions, including negative emotions, are also blunted or toned down [8]. The observed prevalence of anhedonia in patients in the acute phase of depression in this study is consistent with previous reports showing this symptom to be present in approximately 75% of patients [28]. Anhedonia is also a common residual symptom in patients with MDD [29]. While anhedonia was more commonly reported in patients in the acute phase of depression than in those in remission in the present study, just over half of patients who were considered to be in remission continued to report this symptom.

## Methodologic considerations

Research methods such as those described in this study are increasingly used to evaluate the patient experience across different disease areas. Use of an internet-based survey enables the acquisition of a large sample size across different countries over a short period of time and facilitates the collection of data across a relatively large number of variables, permitting detection of cross-sectional response patterns among patient groups.

The main strengths of this study are that it provides information on the experience of emotional blunting in depression from the patient’s own perspective in a large sample, using a well-validated, specific, and detailed assessment tool—the ODQ [16, 17]. To our knowledge, this is the first study to assess the phenomenology of emotional blunting in patients with depression in both the acute and remission phases of the disease. The importance of addressing patient perspectives when managing mental health disorders such as depression is well recognized [30–33].

By design, all patients participating in this study reported that they were experiencing emotional blunting. Further study limitations include the potential for selection and/or recall bias (e.g., patients experiencing emotional blunting who did not feel that their condition was being adequately treated may have been more likely to consent to study participation), lack of information on comorbid conditions that might also have influenced patient responses, and the fact that the patient experience of emotional blunting may have been influenced by the antidepressant treatment(s) received. It should also be noted that all patients in this study were receiving treatment with antidepressant medication; in clinical practice, however, many patients with MDD who experience emotional blunting will discontinue drug treatment. As such, our findings may underestimate the true prevalence and impact of emotional blunting in MDD, particularly during the remission phase of the disease. Finally, due to the cross-sectional study design, any causal inferences should be interpreted with caution.

## Conclusion

In summary, our findings provide new insights into the severity and perceived causes of emotional blunting in patients with depression in both the acute and remission phases of the disease. Of particular note, just over half of patients with MDD reporting emotional blunting considered this to be caused by their depression, while 45% believed that their antidepressant medication was blunting their emotions. Furthermore, just over one-third of all patients were considering or had already stopped taking their antidepressant because of perceived emotion-related adverse effects. This potential risk of premature treatment discontinuation in patients who are experiencing emotional blunting highlights the need for increased awareness and improved recognition of this common symptom in patients with MDD in order to ensure that antidepressant treatment can be targeted to their clinical needs.

## Supplementary Information

**Additional file 1: Table S1.** Patient responses to the ODQ questionnaire.

## Data Availability

The datasets presented in this article are not readily available given the informed consent provided by survey participants. Requests to access the datasets should be directed to the authors.
