# Supplementary material for: Emotional blunting in patients with depression. Part I: clinical characteristics
Source: Ann Gen Psychiatry. 2022 Apr 4;21:10. doi: 10.1186/s12991-022-00387-1 (PMC8981644; doi:10.1186/s12991-022-00387-1)
Supplement: Supplementary file 1 — Additional file 1: Table S1. Patient responses to the ODQ questionnaire. [file 12991_2022_387_MOESM1_ESM.pdf]

Additional file 1

Table S1 Patient responses to the ODQ questionnaire. Responses for the ‘antidepressant as cause’ domain are shown in Table 3.

	Patient net agreement[a] (%)		
	**All patients (*N* = 752)**	**Acute (*n* = 300)**	**Remission (*n* = 452)**
**General reduction in emotions**			
All my emotions, both ‘pleasant’ and ‘unpleasant’, are ‘toned down’	56	55	57
Unpleasant emotions, such as sadness, disappointment, and upset, feel toned down or different in some way	55	51	57
My emotions lack intensity	58	63*	54
Day-to-day life just doesn’t have the same emotional impact on me that it did before my illness/problem	75	83**	70
My emotions are numbed/dulled/flattened compared to before I developed my illness/problem	72	77**	68
**Reduction in positive emotions**			
I don’t fully enjoy things that should give me pleasure, such as beautiful places or things or music	74	85**	66
I don’t look forward to things with eager anticipation	76	85**	70
I don’t have the passion and enthusiasm for life that I should	82	93**	74
I don’t experience pleasant emotions as much as I did before I developed my illness/problem	79	90**	71
I don’t get as much of a ‘high’ from good things in my life as I did before my illness/problem	79	88**	73

**Emotional detachment from others**			
I care less about other people's feelings than I think I should	48	54*	45
I don't have much sympathy for people	43	46	40
Other people being upset doesn't affect me	34	37	31
I don't react to other people's emotions (such as sadness, anger or upset) as much as I did before I developed my illness/problem	54	57	52
I don't have as much sympathy for other people as I did before my illness/problem	53	57	50
**Not caring**			
Because I don't care so much about things, I'm having problems at home	48	59**	41
I feel 'spaced out' and distant from the world around me	72	83**	65
Because I don't care so much about things, I'm having problems at work or college	42	53**	35
I don't care as much about my day-to-day responsibilities as I did before I developed my illness/problem	63	76**	54
I just don't care as much about things as I did before my illness/problem	68	76**	64

[a]Proportion of patients who selected 'agree' or 'agree a little' in response to the statement

$*p < 0.05$, $**p < 0.01$ for difference between the acute and remission phase groups

ODQ, Oxford Depression Questionnaire
